# Supplementary material for: Comparative Meta-Analysis of the Effect of Concentrated, Hydrolyzed, and Isolated Whey Protein Supplementation on Body Composition of Physical Activity Practitioners
Source: Nutrients. 2019 Sep 2;11(9):2047. doi: 10.3390/nu11092047 (PMC6769754; doi:10.3390/nu11092047)

nutrients

MDPI

Review

Comparative Meta-analysis of the Effect of Concentrated, Hydrolyzed, and Isolated Whey Protein Supplementation on Body Composition of Physical Activity Practitioners

Luis Henrique A. Castro [1,*]; Flávio Henrique S. de Araújo [1]; Mi Ye M. Olimpio [1]; Raquel B. de B. Primo [1]; Thiago T. Pereira [1]; Luiz Augusto F. Lopes [2]; Erasmo B. S. de M. Trindade [3]; Ricardo Fernandes [4]; Silvia A. Oesterreich [1]

[1] Graduate Program in Health Sciences – Federal University of Grande Dourados (UFGD), Brazil; posgraduacaosaude@ufgd.edu.br
[2] Faculty of Health Sciences - Federal University of Grande Dourados / Universitary Hospital of Federal University of Grande Dourados, Brazil; medicina@ufgd.edu.br
[3] Graduate Program in Nutrition – Federal University of Santa Catarina (UFSC), Brazil; ppgn@contato.ufsc.br
[4] Graduate Program in Food, Nutrition and Health – Federal University of Grande Dourados (UFGD), Brazil; fcs.ppgans@ufgd.edu.br
* Correspondence: nutricao.luishenrique@gmail.com; Tel.: +55-67-3410-2325

Supplementary material

- Table S1: Details of the central search strategy adapted to the databases.
- Table S2: Quantitative details of the central search results.
- Table S3: Quantitative detailing of the screening by the eligibility criteria of the central search.
- Table S4: Quantitative detailing of results and search screening for unpublished studies.
- Table S5: Individualized bias risk analysis (ROB 2.0).
- Figure S1. Analysis of sensibility (leave-one-out) on global WMD.

Table S1. Details of the central search strategy adapted to the databases

1) PubMed (active filters: "clinical trial" and "humans") / (active options: "include references"). Date of execution: 2018/12/10.	
Strategy	((((“physic* activity” OR “physic* exercise” OR training OR exercise OR coaching OR “resistance training” OR “resistance exercise*” OR gym* OR fit* OR crossfit OR “weight lifting” OR “non-sedentary” OR athlete)) AND (“whey protein” OR whey OR “protein supplement*” OR “whey supplement*” OR casein OR “casein supplement*” OR “whey intake” OR “casein intake” OR “protein intake” OR “whey concentrate” OR “casein concentrate” OR “concentrate protein” OR “whey isolated” OR “casein isolated” OR “isolated protein” OR “hydrolyzed whey” OR “hydrolyzed casein” OR “hydrolyzed protein” OR milk OR “milk protein” OR soy OR “soy protein”)) AND (muscle* OR “mass gain” OR “muscle* gain” OR “muscular gain” OR “muscle* strength*” OR “muscular* strength*” OR “hypertrophy” OR “lean mass” OR “body composition” OR “lean body mass” OR “lean body tissue” OR “fat-free mass” OR “fat free mass” OR “body weight” OR “body mass” OR “skeletal muscle*”)) NOT (child*[Title] OR pediatric*[Title] OR elder*[Title] OR aged[Title] OR rat*[Title] OR mice*[Title])
2) COCHRANE Trials (active options: "search for similar words" and "include references"). Date of execution: 2018/12/10.	
Strategy	(“physical activity” OR “physical exercise” OR training OR exercise OR coaching OR “resistance training” OR “resistance exercise” OR gym OR fit OR fitness OR crossfit OR “weight lifting” OR “non-sedentary” OR athlete)
AND	(“whey protein” OR whey OR “protein supplement” OR “whey supplement” OR casein OR “casein supplement” OR “whey intake” OR “casein intake” OR “protein intake” OR “whey concentrate” OR “casein concentrate” OR “concentrate protein” OR “whey isolated” OR “casein isolated” OR “isolated protein” OR “hydrolyzed whey” OR “hydrolyzed casein” OR “hydrolyzed protein” OR milk OR “milk protein” OR soy OR “soy protein”)
AND	(muscle OR “mass gain” OR “muscle gain” OR “muscular gain” OR “muscle strength” OR “muscular strength” OR “hypertrophy” OR “lean mass” OR “body composition” OR “lean body mass” OR “lean body tissue” OR “fat-free mass” OR “fat free mass” OR “body weight” OR “body mass” OR “skeletal muscle”)
NOT	(child OR pediatric OR elder OR aged OR rat OR mice)
3. Scopus (active selection filters: "Journals" and "Article") / (active exclusion filters: "Review", "Nonhuman" and "Animals") / (active options: "include references"). Date of execution: 2018/12/10.	
Strategy	((“physic* activity” OR “physic* exercise” OR training OR exercise OR coaching OR “resistance training” OR “resistance exercise*” OR gym* OR fit* OR crossfit OR “weight lifting” OR “non-sedentary” OR athlete) AND (“whey protein” OR whey OR “protein supplement*” OR “whey supplement*” OR casein OR “casein supplement*” OR “whey intake” OR “casein intake” OR “protein intake” OR “whey concentrate” OR “casein concentrate” OR “concentrate protein” OR “whey isolated” OR “casein isolated” OR “isolated protein” OR “hydrolyzed whey” OR “hydrolyzed casein” OR “hydrolyzed protein” OR milk OR “milk protein” OR soy OR “soy protein”) AND (“controlled clinical trial” OR “clinical trial” OR “random* clinical trial” OR “group control*” OR placebo OR “comparative study” OR “cross-over” OR “crossover” OR “crossover” OR “double-blind” OR “factorial”) AND (muscle* OR “mass gain” OR “muscle*

	gain" OR "muscular gain" OR "muscle* strength*" OR "muscular* strength*" OR "hypertrophy" OR "lean mass" OR "body composition" OR "lean body mass" OR "lean body tissue" OR "fat-free mass" OR "fat free mass" OR "body weight" OR "body mass" OR "skeletal muscle*") AND NOT (TITLE-ABS-KEY(child* OR pediatric* OR elder* OR aged OR rat* OR mice*)))
4. SportDiscus (active options: "words related", "full text", "equivalent subjects" e "include references"). Date of execution: 2018/12/10.	
Strategy	(("physical activity" OR "physical exercise" OR training OR exercise OR coaching OR "resistance training" OR "resistance exercise" OR gym OR fit OR fitness OR crossfit OR "weight lifting" OR "non-sedentary" OR athlete) AND ("whey protein" OR whey OR "protein supplement" OR "whey supplement" OR casein OR "casein supplement" OR "whey intake" OR "casein intake" OR "protein intake" OR "whey concentrate" OR "casein concentrate" OR "concentrate protein" OR "whey isolated" OR "casein isolated" OR "isolated protein" OR "hydrolyzed whey" OR "hydrolyzed casein" OR "hydrolyzed protein" OR milk OR "milk protein" OR soy OR "soy protein") AND ("controlled clinical trial" OR "clinical trial" OR "random clinical trial" OR "group control" OR placebo OR "comparative study" OR "cross-over" OR "crossover" OR "cross over" OR "double-blind" OR "factorial") NOT(child OR pediatric OR elder OR aged OR rat OR mice))
5. Web of Science (active options: "include references"). Date: on the day of the search in the other databases, the Web of Science network was in a scheduled maintenance (10h00min GMT at 23h00min GMT). For this reason, this search was done afterwards at 2018/12/14. With the corresponding author can be found the screen of the website, in which we will be grateful to share to whom it may interest.	
Strategy	("physic* activity" OR "physic* exercise" OR training OR exercise OR coaching OR "resistance training" OR "resistance exercise*" OR gym* OR fit* OR crossfit OR "weight lifting" OR "non-sedentary" OR athlete)
AND	("whey protein" OR whey OR "protein supplement*" OR "whey supplement*" OR casein OR "casein supplement*" OR "whey intake" OR "casein intake" OR "protein intake" OR "whey concentrate" OR "casein concentrate" OR "concentrate protein" OR "whey isolated" OR "casein isolated" OR "isolated protein" OR "hydrolyzed whey" OR "hydrolyzed casein" OR "hydrolyzed protein" OR milk OR "milk protein" OR soy OR "soy protein")
AND	("controlled clinical trial" OR "clinical trial" OR "random* clinical trial" OR "group control*" OR placebo OR "comparative study" OR "cross-over" OR "crossover" OR "cross over" OR "double-blind" OR "factorial")
AND	(muscle* OR "mass gain" OR "muscle* gain" OR "muscular gain" OR "muscle* strength*" OR "muscular* strength*" OR "hypertrophy" OR "lean mass" OR "body composition" OR "lean body mass" OR "lean body tissue" OR "fat-free mass" OR "fat free mass" OR "body weight" OR "body mass" OR "skeletal muscle*")
NOT	(child* OR pediatric* OR elder* OR aged OR rat* OR mice*)

Table S2. Quantitative detailing of the central search results

PARTIALS	
**1) PubMed**	**RESULTS (n)**
General search	1.846
After filter "clinical trial"	460
After filter "humans"	457
Total	457
Imported references	18
Total sorted results	475
**2) Cochrane - CENTRAL**	**RESULTS (n)**
Total search	1.118
Imported references	0
Total sorted results	1.118
**3) Scopus**	**RESULTS (n)**
General search	11.018
After filter "Journals"	8.608
After filter "Keyword"	3.850
After filter "Document type"	2.873
Total	2.873
Imported references	31
Total sorted results	2.904
**4) SPORTDiscus**	**RESULTS (n)**
Total search	696
Imported references	0
Total sorted results	696
**5) Web of Science**	**RESULTS (n)**
Total search	106
Imported references	0
Total sorted results	106
**TOTAL**	
**TOTAL NUMBER OF SEARCHES**	**5.250**
**TOTAL OF IMPORTED REFERENCES**	**49**
**TOTAL OF SORTED RESULTS**	**5.299**
**TOTAL OF DUPLICATES**	**1.772**
**TOTAL AFTER DELETING DUPLICATES**	**3.527**

Table S3. Quantitative detailing of the screening by the eligibility criteria of the central search

**1st SCREENING - TITLE**	
Reason for exclusion	*Excluded results (n)*
Inclusion criteria (be a research on whey protein supplementation)	1617
Exclusion criteria 1 (not a research on humans)	13
Exclusion criteria 2 (not a clinical scientific article)	217
Total after the first screening	1680
**2nd SCREENING - ABSTRACT**	
Reason for exclusion	*Excluded results (n)*
Inclusion criteria (be a randomized clinical trial)	188
Exclusion criteria 1 (the population studied is children, adolescents or the elderly)	967
Exclusion criteria 2 (the population studied has some pathology)	361
Total after the second screening	164
**3rd SCREENING – FULL TEXT**	
Reason for exclusion	*Excluded results (n)*
Inclusion criteria (have assessed physical activity)	05
Exclusion criteria (absence of control group or placebo/ full cross-over)	21
Not being a clinical research	7
Not be a research about whey	11
Sick population	04
Age group	01
Not-human	01
Preliminary selection	114
**FINAL SCREENING**	
Inclusion criteria not filled by the studies	*Excluded results (n)*
Analysis of fat free mass and fat mass	73
Analysis of body composition by gold-standard methods	10
Whey protein intervention compared to other food kinds	13
Absence of variables parallel to the listed interventions	1
Presence of at least one comparison group not related to control and/or placebo	3
Studies without multiple publications	4
**Final selection**	**10**

Table S4. Quantitative detailing of results and search screening for unpublished studies

**DATABASE***	**RESULTS (n)**
NIH U.S National Library of Medicine (Clinical Trials)	304
Registros Brasileiros de Ensaios Clínicos (REBEC)	5
International Clinical Trials Registry Platform (WHO/ICTRP)	331
Total	640
**SCREENING**	**RESULTS (n)**
1. Protocols derived from previous records	113
2. Protocols screened by title	527
2.1 Excluded	256
3. Protocols screened by further information available	271
3.1 Excluded by the eligibility criteria (1-14)	266
4. Protocols checked for availability of preliminary data	4
4.1 Excluded due to data unavailability	4
Total of protocols available for inclusion in the review and/or meta-analysis	0

*Search executed on 2018/12/14.

Table S5. Individualized bias risk analysis (ROB 2.0)

**RANDOMIZED CLINICAL TRIAL**	**BIAS DOMAIN**				
	1	2	3	4	5
Burke et al [31]	Low risk	Some concerns	Low risk	Low risk	Low risk
Cribb et al [32]	Low risk	Low risk	Low risk	Low risk	Low risk
Herda et al [33]	Some concerns	Low risk	Low risk	Low risk	Some concerns
Hulmi et al [34]	Low risk	High risk	Low risk	Low risk	Low risk
Lockwood et al [35]	Some concerns	Some concerns	Low risk	Some concerns	Some concerns
Naclerio et al [36]	Some concerns	Some concerns	Low risk	Some concerns	Low risk
Naclerio et al [37]	High risk	Some concerns	Some concerns	Some concerns	Low risk
Reidy et al [38]	Some concerns	Low risk	Low risk	Some concerns	Some concerns
Taylor et al [39]	High risk	Some concerns	Low risk	Low risk	Some concerns
Volek et al [40]	High risk	Low risk	Low risk	Low risk	Some concerns

1 - Bias resulting from the randomization process; 2 - Bias due to deviations from the intended interventions; 3 - Bias due to lack of outcome data; 4 - Bias in the measurement of the result; 5 - Bias in the selection of the reported result.

Figure S1. Analysis of sensibility (leave-one-out) on global WMD

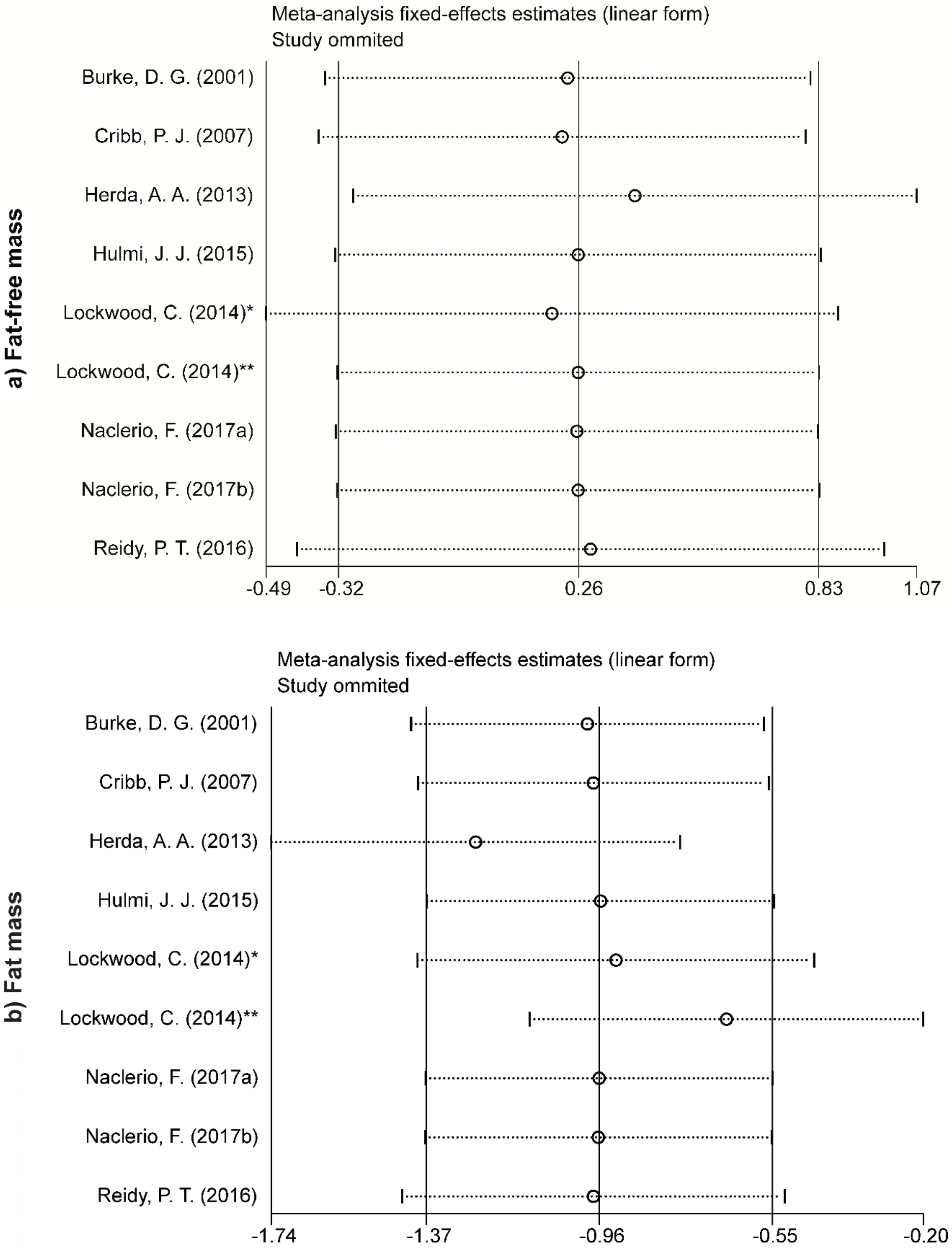

Supplement: Supplementary file 1 [file nutrients-11-02047-s001.pdf]
